# QTL mapping and improvement of pre-harvest sprouting resistance using *japonica* weedy rice

**DOI:** 10.3389/fpls.2023.1194058

**Published:** 2023-06-05

**Authors:** Chang-Min Lee, Hyun-Su Park, Man-Kee Baek, O-Young Jeong, Jeonghwan Seo, Suk-Man Kim

**Affiliations:** ^1^ Crop Breeding Division, National Institute of Crop Science, Rural Development Administration, Wanju, Republic of Korea; ^2^ Department of Ecological & Environmental System, Kyungpook National University, Sangju, Republic of Korea

**Keywords:** rice, pre-harvest sprouting (PHS), quantitative trait loci (QTL), germination, dormancy

## Abstract

The stability of cultivation and production in terms of crop yield has been threatened by climate change due to global warming. Pre-harvest sprouting (PHS) is a threat to crops, especially staple foods, including rice, because of reductions in yield and quality. To address the problem of precocious germination before harvest, we performed quantitative trait loci (QTL) analysis for PHS using F_8_ RILs populations derived from *japonica* weedy rice in Korea. QTL analysis revealed that two stable QTLs, *qPH7* and *qPH2*, associated with PHS resistance were identified on chromosomes 7 and 2, respectively, explaining approximately 38% of the phenotypic variation. The QTL effect in the tested lines significantly decreased the degree of PHS, based on the number of QTLs included. Through fine mapping for main QTL *qPH7*, the region for the PHS was found to be anchored within 23.575–23.785 Mbp on chromosome 7 using 13 cleaved amplified sequence (CAPS) markers. Among 15 open reading frames (ORFs) within the detected region, one ORF, Os07g0584366, exhibited upregulated expression in the resistant donor, which was approximately nine times higher than that of susceptible *japonica* cultivars under PHS-inducing conditions. *Japonica* lines with QTLs related to PHS resistance were developed to improve the characteristics of PHS and design practical PCR-based DNA markers for marker-assisted backcrosses of many other PHS-susceptible *japonica* cultivars.

## Introduction

As an important global staple food crop, rice (*Oryza sativa* L.) has played a key role in providing calories and protein in enormous production efforts (Wei et al., 2013). Under the current threat of global warming and a growing population, rice production is expected to increase (Sohn et al., 2021). These environmental and demographic factors threaten the practical stability of rice cultivation and yield, raising concerns regarding the role of rice as a staple food crop. In particular, changes in rainfall patterns caused by climate change have resulted in pre-harvest sprouting (PHS), giving rise to a deterioration in both grain quality and yield (Liu et al., 2013; Shu et al., 2015).

PHS is the precocious germination of grains before harvest. The premature germination of grains within panicles in paddy fields occurs when crops are exposed to humid weather conditions, favorable for germination, during the grain ripening stages (Hanumanthappa et al., 2016; Park et al., 2021). Therefore, PHS is a serious global agricultural issue in terms of both production and economics and requires urgent preventive measures (Tai et al., 2021). Xu et al. (2019) reported that PHS causes a yield loss of approximately 6% to 20% hybrid rice yield in affected fields on average. In the case of wheat, Miao et al. (2013) reported that PHS leads to a yield loss of approximately 10%, with the average annual loss caused directly by PHS exceeding $1 billion worldwide (Bewley et al., 2006).

Seed dormancy is an important trait that determines the degree of PHS, which has a positive or negative effect on plant germination. Physical factors, such as the tight packing of the caryopsis and the seed tissue that covers it (hull and pericarp), play a crucial role in inhibiting seed germination by restricting the diffusion of oxygen and water to the embryo. This tight physical barrier prevents both imbibition and the emergence of the radicle, resulting in dormancy (Gu et al., 2004; Barik et al., 2017). In addition to physical factors, endogenous hormones, such as gibberellic acid (GA) and abscisic acid (ABA), also contribute to seed dormancy by controlling the balance between germination and dormancy (Gu et al., 2003; Finkelstein et al., 2008; Chen et al., 2021). Enhanced ABA biosynthesis promotes seed dormancy, while a decrease in ABA content or blocking of ABA signaling results in reduced seed dormancy. In contrast, seed germination is promoted by GA, which not only initiates embryo activity but also breaks the seed coat surrounding it (Chen et al., 2021). The balance of these phytohormones is crucial in the antagonistic regulation of seed dormancy and germination in many plant species (Shu et al., 2015). Furthermore, some studies have reported the role of ethylene in breaking dormancy (Matilla, 2000; Corbineau et al., 2014). Nevertheless, for cereals, including rice, a moderate degree of dormancy is desirable to prevent PHS (Gu et al., 2004). Dehulled seeds recorded more germination than seeds with intact hulls, acting as a barrier to the diffusion of oxygen to the embryo (Baek and Chung, 2014). Pericarp impermeability to oxygen can cause seed dormancy in ABA, short-chain saturated fatty acids, and several of these factors (Bewley and Black, 1985; Hayashi, 1987). In particular, ABA has shown that endospermic sugars exert their action as a basal energy source for seed germination, imposing seed dormancy and germination *via* their influence on ABA signaling (Du et al., 2018).

Seed dormancy is a complex quantitative trait that is controlled by environmental and endogenous genetic factors, and has the largest impact on PHS resistance. In recent years, PHS research has focused on identifying the individual genes that impact dormancy (Vetch et al., 2019). Several quantitative trait loci (QTLs) controlling seed dormancy have been identified in rice and other cereal crops, including the model plant Arabidopsis. In rice, 185 QTLs have been detected on all 12 chromosomes of the rice genome (Sohn et al., 2021). Recently, five QTLs were detected using a recombinant inbred line (RILs) derived from a *japonica* cross on chromosome 3, 4, and 11 (Cheon et al., 2020). *qPHS1-1* and *qPHS1-2* were identified repeatedly within the same regions on chromosome 1, under both growth chamber and field conditions (Jang et al., 2020). In addition, *qSDR9.1* and *qSDR9.2* on chromosome 9 were identified using advanced backcross lines with SSR markers (Mizuno et al., 2018). As a novel approach, genome-wide association (GWA) studies have been conducted on PHS and seed dormancy in rice. In one study, two loci associated with PHS were detected on chromosome 1 and 4 in 277 accessions using 296 K single nucleotide polymorphisms (SNPs) (Lee et al., 2021). Novel significant SNPs were found to explain 34.9% of the phenotypic variation identified on chromosome 8 in an indica-only population consisting of 453 accessions genotyped using 5,291 SNPs (Lu et al., 2018). Association analysis revealed 16 loci significantly associated with seed germination percentage in freshly harvested seeds and 38 in after-ripened seeds, using 350 accessions from *indica*, *japonica*, and *Aus.* germplasm (Magwa et al., 2016). Only the molecular cloning of a few genes, namely *SD1-2* and *Sdr4*, has been reported as a regulator of seed dormancy by map-based cloning (Sugimoto et al., 2010; Ye et al., 2015). Although several QTLs associated with seed dormancy and PHS have been reported in diverse germplasms, including weedy and wild rice, the molecular mechanisms of dormancy release and PHS remain unclear.

To overcome the damage caused by PHS due to climate change as a result of global warming change, we constructed a rice mapping population from a novel genetic source with PHS tolerance. In this study, QTL was analyzed using RILs and find-mapping was performed with the next-generation sequencing (NGS) dataset to evaluate the PHS resistance of *japonica-*type Korean weedy rice, Wandoaengmi6. Then, a PCR-based DNA marker was developed for marker-assisted breeding and the expression of candidate mRNA levels associated with PHS resistance in weedy rice was evaluated. The corresponding results provide a practical reference for use in abiotic stress breeding programs on climate change by the development of cultivars with moderate dormancy using a marker-assisted backcross approach.

## Materials and methods

### Plant materials and mapping population

Plant materials and mapping population Hwayeong, a mid-maturing *japonica* cultivar with high PHS, and Wandoaengmi6, a *japonica*-type Korean weedy rice with tolerance to PHS, were crossed to develop RILs population using a single descent method (SSD). The population (F_8_), composed of 186 lines, was used to assess the phenotypic data of PHS and to construct a molecular genetic map to identify the QTLs controlling resistance sprouting for two years (2019-2020).

### Evaluation of pre-harvested sprouting

The parents and RILs were cultivated in an experimental field at the National Institute of Crop Sciences (NICS), Rural Development Administration (RDA), Wanju, South Korea, in 2019 and 2020. Seeds were sown in early May and seedlings were transplanted into paddy fields in early June. The plants in each row were spaced 15 cm apart, and the rows were spaced 30 cm apart. Plants were cultivated and evaluated according to the standard evaluation method for rice (RDA, 2012). To score the level of resistance to PHS, three panicles of parents and 186 RILs were harvested at 40 DAH (days after heading), which means meeting the condition at the same time that the cumulative temperature reached 1,000°C after heading in 2019 and 2020. The harvested panicles were immediately stored at 25°C and 100% humidity. After seven days of incubation, the number of germinated seeds per panicle was counted. The PHS rate (%) was calculated as follows: (germination seeds/total filled seeds) × 100%. The average germination percentage was calculated for each line.

### Genotyping and linkage mapping

SNPs showing polymorphic patterns within the parents were confirmed using the 7 K Infinium SNP genotyping platform (Illumina^®^) at the Genotype Service Laboratory of the International Rice Research Institute (IRRI) (Park et al., 2019). The SNPs selected from the genotypic data sets decoded to generate SNP data using GenomeStudio software were used to construct a linkage map using QTL IciMapping (version 4.0) (**Supplementary Figure S2**) (Meng et al., 2015). The mapping distance during linkage map construction was calculated using the Kosambi mapping function, and the options By LOD and By Input were used for the grouping and ordering of the selected factors, respectively.

### Data analysis

The study utilized both phenotypic and genotype data, including SNPs, to detect QTLs associated with PHS resistance in the population. We scored the PHS rate as <5%, 5-15%, 15-25%, 25-35% and >35% as 1, 3, 5, 7, 9 degrees, respectively. Permutation tests with 1,000 replicates (*P* ≤ 0.05) were applied to confirm significant threshold values of the limit of detection LOD scores for QTL detection, according to the literature (Churchill and Doerge, 1994). The QTLs were named in accordance with the method proposed by McCouch and the Committee on Gene Symbolization, Nomenclature and Linkage, Rice Genetics Cooperative (McCouch, 2008).

### Development of DNA markers using whole-genome resequencing

For fine mapping, additional DNA markers were used to design new primers using WGR within the target region. WGR was performed using an Illumina NovaSeq 6000 system (Illumina, USA) following the protocol for 2 × 100 sequencings. The DNA library was prepared according to the Truseq Nano DNA library preparation protocol (cat. no. FC-121-4001). Using the resequencing data, CAPS markers and specific restriction sites in the target region were designed using the marker design software RICE-SNP-MINER.

### qPCR analysis

The real-time PCR array was performed on an Exicycler™ 384 Real-Time Quantitative Thermal Block (Bioneer, Daejeon, Korea) with the following cycling parameters: 40 cycles of 95°C for 5 s, 58°C for 25 s, and 72°C for 30 s. Data analysis was based on the relative quantitative method, and the ΔΔCT value was used to determine the relative fold-change in expression. All data were normalized to the expression level of the reference gene Os03g08010(*eEF1*-a).

## Results

### Evaluation of PHS using the RIL population

**Figure 1:**
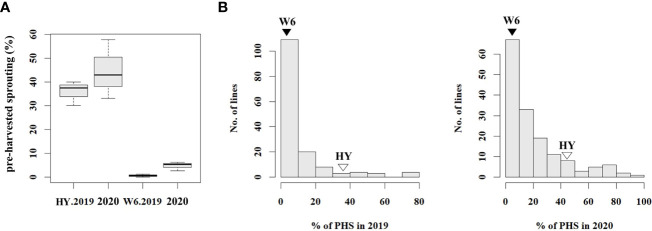
Distribution of pre-harvested sprouting (PHS) percentages in tested plant materials. **(A)** Box plot showing PHS percentages for the parents in 2019 and 2020. **(B)** Frequency distribution of PHS percentages in RIL population derived from a cross between Hwayeong and Wandoaengmi6, for the years 2019 and 2020. The values for Hwayeong (HY) and Wandoaengmi6 (W6) are indicated above the unfilled and filled triangles, respectively. The data provides insight into the variation in PHS percentages across different plant materials and years.

To analyze the phenotype of the population used for resistance to PHS, PHS rates were evaluated using 186 RILs and the parents, Hwayeong and Wandoaengmi6, from 2019 to 2020. The average PHS rates of Hwayeong and Wandoaengmi6 were 40.2 ± 9.73% and 2.7 ± 2.60%, respectively (**Figure 1A** and **Supplementary Figure S1**). The degree of PHS in the population ranged from 0 to 92.2% and did not appear to exhibit a normal distribution curve for the tested trait (**Figure 1B**). The distribution of the PHS for two years was biased towards resistance and showed positive skewness (1.20) and leptokurtosis (1.3).

Next, the segregation of PHS was evaluated to understand the inheritance pattern (**Supplementary Table S1**). Based on the reference point (RF), the segregation ratio was the phenotypic ratio of 9:3:4 (R:M:S) at RF-III and 3:1 (R:S) at RF-II (*P* < 0.01). Considering the advanced generation of the population, the inheritance pattern suggested that multiple genes, rather than a single gene, would dominate the tested trait.

To confirm the dormancy of seeds related to PHS, the germination of panicles, intact seeds, and de-hulled seeds of the parents was evaluated at 40 and 60 days after heading (DAH) (**Table 1**). At 40 DAH, the germination rate of the panicle and intact seed of Wandoaengmi6 was less than 5%, while that of the de-hulled seed was similar to that of Hwayeong. In addition, at 60 DAH, the germination rates of panicles, intact seeds, and de-hulled seeds of Hwayeong were >95%. In the case of Wandoaengmi6, the germination rate of the panicle and the intact seeds of Wandoaengmi6 were almost 6%, maintaining dormancy, while seed dormancy disappeared under de-hulled seed conditions.

**Table 1:** Difference in germination rate of panicles, intact seeds, and de-hulled seeds.

Parents	% of germination at 40 DAH^a^			% of germination at 60 DAH^b^		
	Panicle	Intact seed	De-hulled seed	Panicle	Intact seed	De-hulled seed
Hwayeong	40.2 ± 9.73	39.9 ± 6.41	77.9 ± 3.52	96.1 ± 1.74	95.9 ± 3.92	97.0 ± 1.47
Wandoaengmi6	2.7 ± 2.60	3.0 ± 3.96	78.1 ± 16.19	6.4 ± 2.58	6.8 ± 7.19	80.0 ± 8.74

^a)^ Forty days after heading.

^b)^ Storage for additional 20 days at room temperature after harvest at 40 DAH.

The differences were evaluated according to the elapsed time after heading.

### QTL analysis for PHS

To identify the genetic loci associated with PHS in the population, a linkage map was constructed and QTL analysis was performed using inclusive composite interval mapping (ICIM) analysis. Among the 7098 SNPs tested, 1024 SNPs showed polymorphic patterns between the parents (**Supplementary Table S2**). A total of 368 SNPs were selected to configure the linkage map after removing markers that were stacked in the same location or showed separation distortion, which was performed using *X^2^*-test goodness of fit for the expected allelic frequency of 1:1 confirmed at *P* < 0.01.

**Figure 2:**
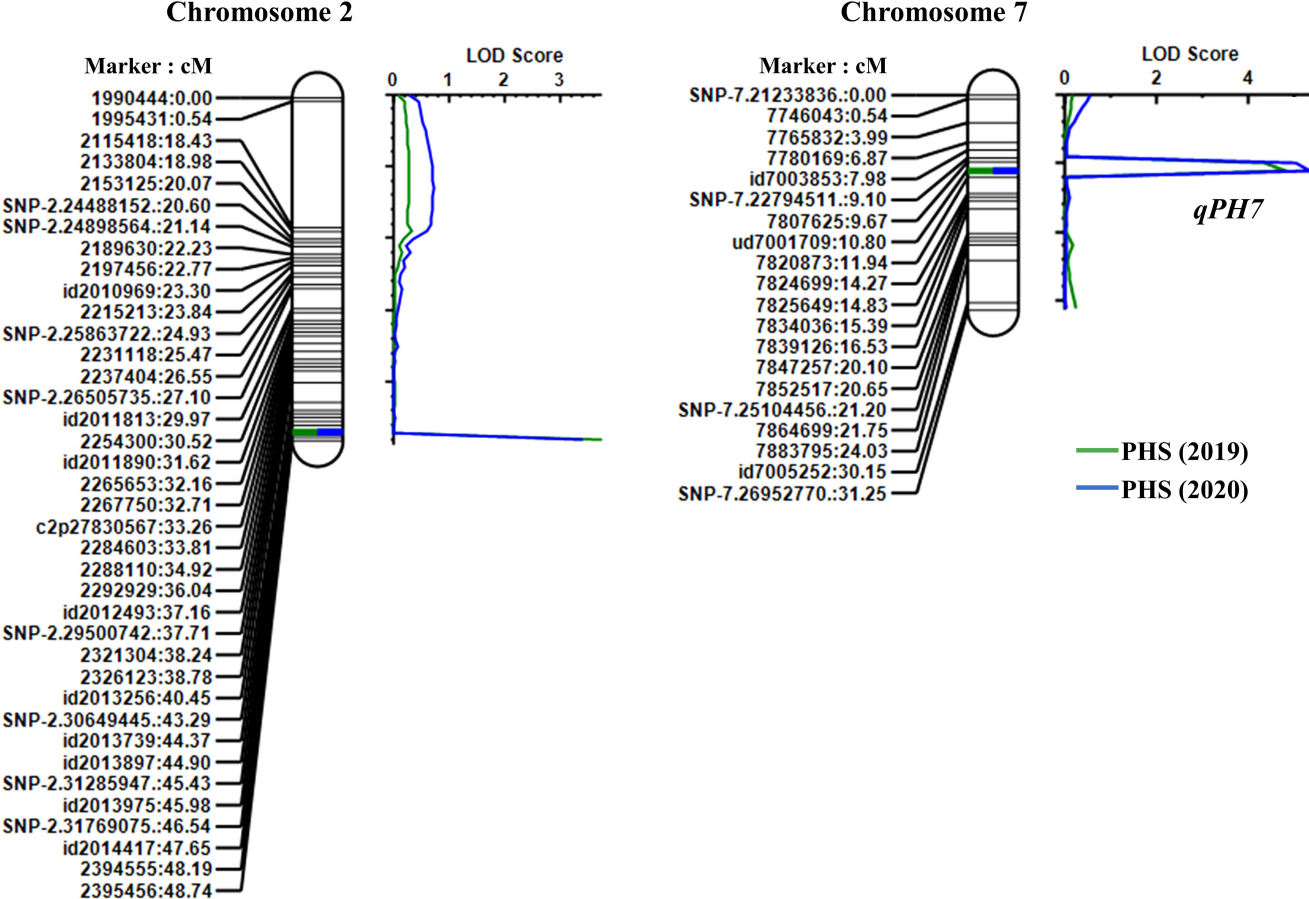
Location of QTLs for PHS resistance detected in the linkage map on chromosomes 2 and 7. Marker names and linkage distances (cM) are shown on the left of each chromosome, and each QTL is presented to the right of the linkage map.

From the results of the QTL analysis, two QTLs were repeatedly detected on chromosome 2 and 7 during both years (**Figure 2**). One QTL, *qPH7*, with an LOD score of 4.26 (2019) and 4.43 (2020), was the allele derived from Wandoaengmi6 and detected within ud7001709 and 7820873 on chromosome 7, explaining 17.49% (2019) and 19.13% (2020) of phenotypic variation (*R^2^*) in ICIM analysis (**Table 2**). Another QTL, *qPH2*, with a LOD of 3.57 (2019) and 3.27 (2020), was detected within flanking markers (id2014417 and 2394555) on chromosome 2 with an *R^2^* of 14.52% (2019) and 13.78% (2020), respectively. The allele of *qPH2* was confirmed to have been derived from Hwayeong (**Table 2**).

**Table 2:** QTLs for pre-harvested sprouting from a recombinant inbred line (RIL) population.

QTLs	Chr.^a^	Flanking markers		Year	LOD	PVE^b^ (%)	Add^c^
		Center	Right				
*qPH2*	2	id2014417	2394555	2019	3.57	14.52	−1.00
				2020	3.27	13.78	−0.92
*qPH7*	7	ud7001709	7820873	2019	4.26	17.49	1.10
				2020	4.43	19.13	1.08

^a)^ Chromosome number.

^b)^ Percentage of phenotypic variation explained by QTL.

^c)^ Additive effect.

QTLs were derived from a cross between Hwayeong × Wandoaengmi6.

### Effect of detected QTLs

**Figure 3:**
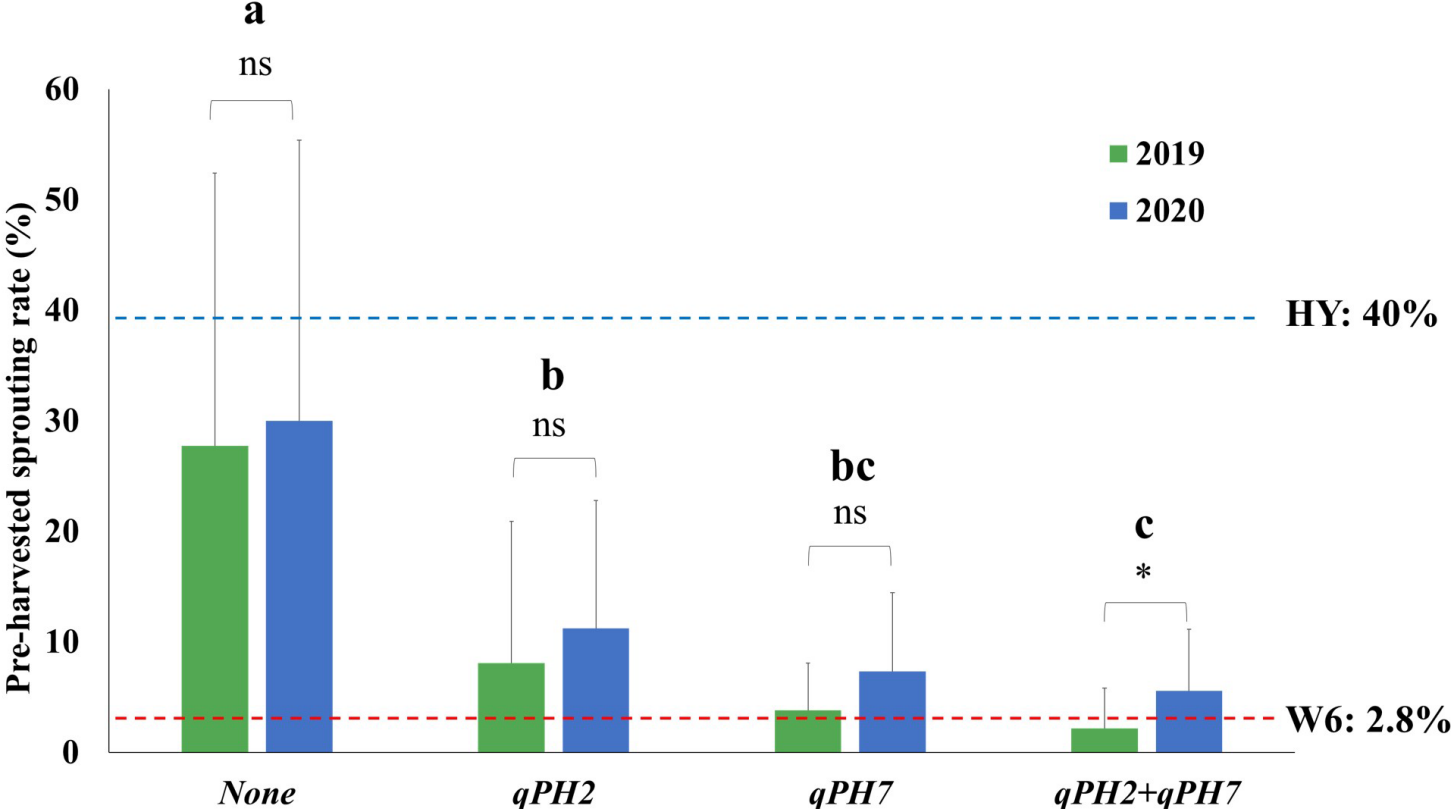
Comparison of QTL effects in the tested RILs under PHS-inducing conditions for seven days. The four groups (none, *qPH2*, *qPH7*, and *qPH2+qPH7*) divided by QTLs were tested to verify the QTL effects on PHS in the population for two years (2019-2020). Phenotypic variation in each group for the tested years was evaluated using Student’s t-test P < 0.05; *: significant difference (P < 0.05); ns: no significant difference (*P* > 0.05). The significant difference in the rate of PHS among each group was analyzed using Duncan’s multiple range test (DMRT). a, b, c : Means followed by the same letter are not significant at the 5% significance level. HY, Hwayeong; W6, Wandoaengmi6.

The QTL effect detected in this study was assessed by comparing the difference in the PHS rate in the population according to the number of QTL detected under the PHS-inducing condition (**Figure 3**). The mean PHS rate of lines with two QTLs (*qPH7* + *qPH2*) was 3.8 ± 4.6% at seven days after treatment (DAT) under these conditions, showing the difference in the mean PHS rate between 2019 and 2020. In the case of the two single QTLs, *qPH7* and *qPH2* had the mean PHS rate of 5.5 ± 5.7% and 9.6 ± 12.2%, respectively, with no significant difference from the mean PHS rate between both years. The rate in lines with no QTLs was 28.8 ± 25.0%, indicating susceptibility to PHS like Hwayeong. Resistance to PHS was significantly improved in the QTL-introgression lines, especially in single QTL, *qPH7*, and the QTL combination, *qPH7 + qPH2*.

### Dissection of the target region of *qPH7*

**Figure 4:**
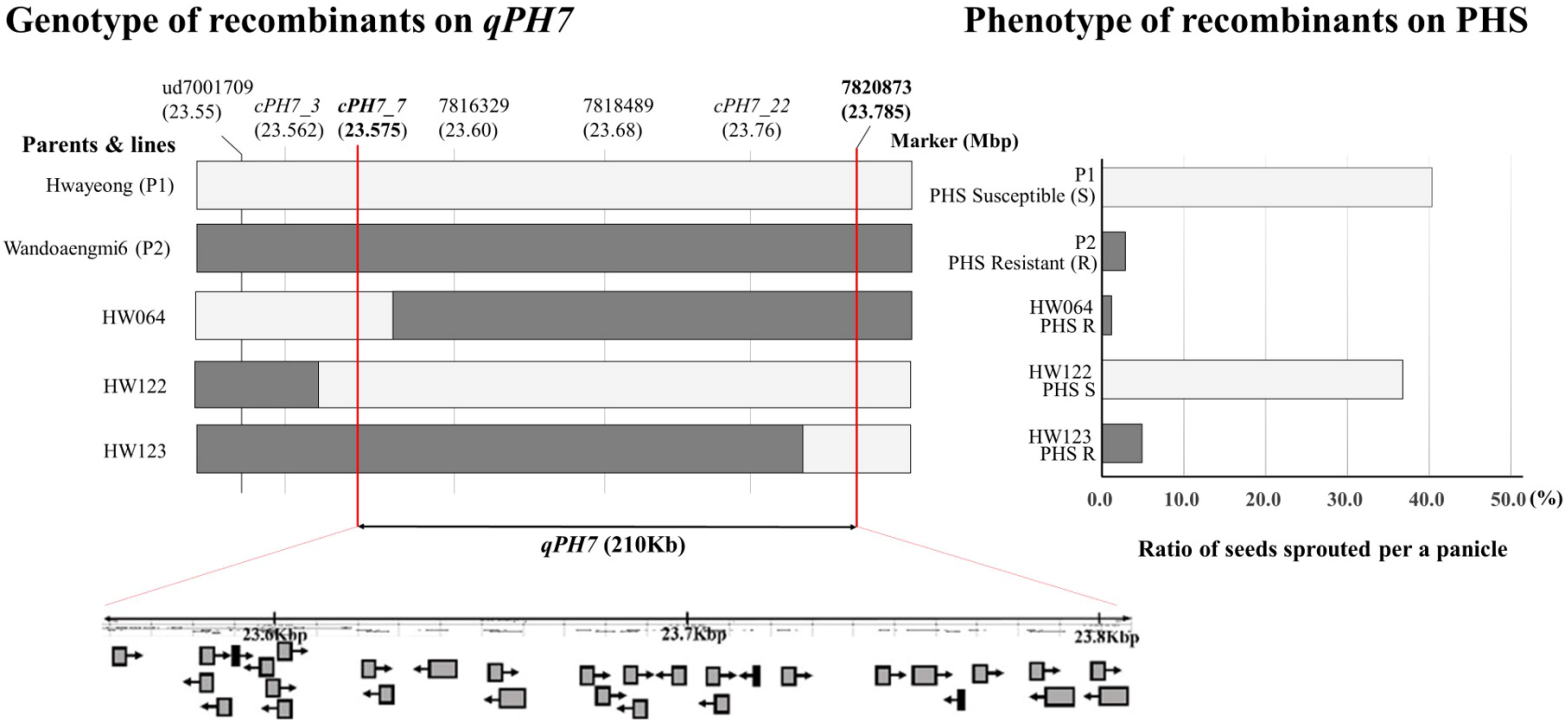
Identification of target regions for pre-harvested sprouting by graphical dissection of HW064, HW122, and HW123. White and dark bars in the graph indicate allele patterns derived from Hwayeong and Wandoaengmi6, respectively. The graph on the right shows the pre-harvested sprouting (%) of the parents and the three selected recombinants. P1 and HW122 show the same phenotype with relatively high PHS rates, whereas P2, HW064, and HW123 had low PHS rates, and all recombinants have no *qPH2*.

Fine mapping of *qPH7* on chromosome 7 was performed using recombinant lines and additional marker sets developed based on next-generation sequencing (NGS) data. We selected three recombinant plants (HW064, HW122, and HW123) by assessing the discordance between phenotype and genotype in the target region defined by ud7001709 to 7820873 on chromosome 7 (**Figure 4**). Among the 23 cleaved amplified sequence (CAPS) markers designed for QTL dissection, 13 markers showed a polymorphic pattern in a parental survey and were anchored in order in the region within 23.575–23.785 Mbp on chromosome 7 (**Supplementary Table S3**). According to the results, the target region was rearranged as approximately 210-Kbp segments delimited by the markers cPH7_7/EcoR I and 7820873. While another three markers, namely 7816329, 7818489, and cPH7_22, were placed in the region, no recombination events were observed. A total of 28 open reading frames (ORFs) based on OS-Nipponbare-Reference-IRGP 1.0 were identified in the rearranged target region (**Supplementary Table S4**). ORFs identified in the detected range were listed as candidate genes conferring resistance to PHS in the population. Eight ORFs encoded proteins of the binding domain and seven ORFs were related to hypothetical proteins or genes. Six ORFs had the solved structure of a homologous protein, and four ORFs encoded functional proteins. Two other ORFs encoded transcription factors, while one ORF encoded a non-protein-coding transcript.

### Expression of genes associated with PHS

**Figure 5:**
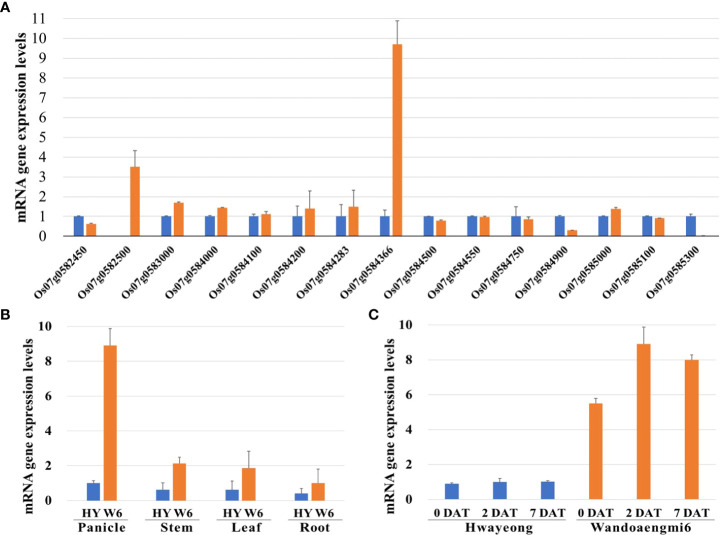
qPCR analysis of gene expression patterns in PHS using parents, Hwayeong (HY) and Wandoaengmi6 (W6). **(A)** mRNA expression levels of 19 open reading frames (ORFs) in the target region using panicles from the parent at 2 DAT. **(B)** mRNA expression levels of Os07g054366 in the parent at three time points (0, 2, and 7 DAT). **(C)** Comparison of mRNA expression levels of four different parts of the parent, which were collected at 2DAT.

The expression levels of all the ORFs detected in the target region were evaluated using primer sets designed based on the coding sequence (CDS) of each ORF. Expression in the panicles of the parents at 2 DAT under PHS-inducing conditions was screened by qPCR analysis of the identified candidates. Of the 28 candidates, 15 ORFs were amplified by qPCR (**Figure 5A**). Among these, one ORF, Os07g0584366, exhibited upregulated expression levels in Wandoaengmi6, which were approximately nine times higher than that of Hwayeong. Meanwhile, the upregulation of the ORF was mainly found in the panicles of Wandoaengmi6 (**Figure 5B**). In addition, gene expression was maintained at a constant level in the panicle regardless of the timing of treatment (**Figure 5C**).

### Development of *japonica* lines with improved resistance to PHS

To improve the resistance to PHS in *japonica* cultivars, we analyzed the genotypic and phenotypic data of the RILs obtained in this study. Performing validation tests for *qPH7* and *qPH2*, 30 lines were selected, including two QTLs, and compared with major agronomic traits, along with blast resistance and grain traits (**Table 3**; **Supplementary Tables S3, S5, S6**). Three lines, HW20, 23, and 36, were found to show similar traits and improved resistance to blast and PHS.

**Table 3:** Comparison of agronomic performance and pre-harvested sprouting of parents and three selected lines, including *qPH7*.

Parents and lines	Major agronomic traits^a^				Germination		Blast resistance (WJ, GH^d^)	QTLs^e^ (*qPH7, qPH2*)
	DH (day)	CL (cm)	PL (cm)	NP	PHS (%)	Nor^c^ (%)		
Hwayeong	92ab	79.6c	20.8ab	12.4a	44.1a	96.5a	S	−, +
Wandoaengmi6	92a	103.4a	19.8bc	11.8ab	5.0b	98.0a	R	+,−
HW20	87a	78.4bc	21.6a	9.2bc	1.3b	100a	MR	+,+
HW23	87a	82.2c	18.0d	9.0c	3.1b	97.5a	R	+,+
HW36	90a	85.6b	19.2cd	12.6a	1.2b	71.5b	R	+,+

^a)^ DH, days to heading; CL, culm length; PL, panicle length; NP, number of panicles; PHS, pre-harvested sprouting.

^b)^ Duncan multiple range test (DMRT) of agronomic traits in the tested lines. Means followed by the same letter are not significant at the 5% level.

^c)^ Nor, germination under normal conditions ( 25°C and 14 days).

^d)^ WJ, Wanju; GH, Gyehwa test field.

^e)^ ‘+’ is present in QTL, and ‘−’ is absent.

## Discussion

In rice, pre-harvest sprouting can lead to a reduction in grain yield and quality, and adequate dormancy is important for resistance to PHS (Gu et al., 2004; Sohn et al., 2021). In this study, we aimed to improve the PHS resistance of *japonica* cultivars in response to increasing PHS damage caused by climate change, using the *japonica*-type Korean weedy rice, Wandoaengmi6, as a donor with high PHS resistance after harvest.

In the phenotypic assay for PHS, the parents Hwayeong and Wandoaengmi6 were found to be positive and negative for the assay conditions of PHS 40 DAH, respectively. In the case of Wandoaengmi6, seed dormancy was maintained under both intact seed and panicle conditions, which was broken 40 DAH, while the dormancy was disappeared in de-hulled seeds (**Table 1**). These results suggest that the PHS resistance of Wandoaengmi6 was caused by coat-imposed dormancy. It is generally known that intact seeds show higher dormancy than de-hulled seeds (Hanumanthappa et al., 2015; Hanumanthappa et al., 2016). The tight packing of the caryopsis inside the hull has been accepted as the main factor controlling seed dormancy, preventing the diffusion of oxygen and water into the embryo (Barik et al., 2017).

To evaluate the inheritance pattern of resistance derived from Wandoaengmi6, genetic analyses were performed over two years using the population tested in this study (**Figure 1**). Although the distribution was found to be biased relative to the resistance, the segregation ratio did not fit the expected ratio (e.g. 3:1) controlled by a single gene (**Supplementary Table S1**). In previous studies, seed dormancy has been treated as a quantitative trait controlled by polygenes, with effects regulated by environmental factors and genetic background, as reported in many crops, including rice (Anderson et al., 1993; Lin et al., 1998; Lijavetzky et al., 2000; Shao et al., 2018). To identify QTLs associated with PHS resistance, we developed RILs and used them as the mapping population for linkage analysis. Two significant QTLs were identified in the population using ICIM analysis, namely *qPH2* (PVE of 14.5%) and *qPH7* (PVE of 18.3%) (**Figure 2**). In addition, epistatic analysis was performed to detect interactions between QTL-QTL or QTL-background marker loci; however, no epistatic interactions were observed in this population. To date, several genetic studies based on molecular markers for the traits, such as PHS, seed dormancy, and low-temperature germination, have been carried out and 185 QTLs have been identified and reported in rice 12 chromosomes, mainly concentrated in chromosome 1, 3, and 7 (Sohn et al., 2021). Among these, there have been several meaningful reports related to PHS on chromosome 7, as with *qPH7* in this study. Dong et al. (2003) reported that *qPHS-7* on the long arm of chromosome 7 contributed to PHS resistance from IR24, explaining 22.0% of PVE. Similarly, Li et al. (2011) reported that the QTL *qDGR7* derived from the allele of *indica* cv. Minghui63 strongly influenced seed dormancy. The QTLs *qSD^s^-7-1* and -*7-2* enhanced the seed dormancy of weed rice SS18-2, which was tightly linked to the red pericarp color gene *Rc* (Gu et al., 2004). *qMT-SGC7.2* was identified for seed germination capability using various storage methods (Jiang et al., 2011). Wang et al. (2014) also reported one major QTL, *qSD7.1*, for seed dormancy derived from IR26, which was tightly linked to heading date in rice.

Considering the sum of PVE on two QTLs (approximately 32.5%), we tested the effect of QTL combinations using the QTLs detected in this study (**Figure 3**). With QTLs *qPH7* and *qPH2*, lines with *qPH2* showed relatively weak resistance compared to the PHS donor; however, lines including every individual QTL showed resistance to PHS. Lines with both mutations showed the strongest resistance within the lines. However, the effect of QTL combinations (*qPH7* + *qPH2*) was not significant compared to the effects of a single QTL *qPH7* or the donor. This was attributed to the interactions between genetic backgrounds by both parents or other alleles associated with PHS resistance that was not detected in the study.

Since *qPH7* was associated with PHS resistance, fine mapping was performed to the target region anchored by the flanking markers ud7001709 and 7820873 on chromosome 7 (**Figure 4**). Additionally, CAPS markers were also developed based on the NGS data of the parents (**Supplementary Table S3**), and the target region was narrowed down by approximately 210 Kbp using three recombinants, namely HW064, HW122, and HW123. The results revealed that the physical position of *qPH7* was reduced from 23.575 Mbp to 23.785 Mbp. According to previous studies, the position near the target region of *qPH7* on the long arm of chromosome 7 seems to be concentrated in QTL/genes associated with PHS resistance. Indeed, five QTL/genes, including *Sdr4*, which is a seed dormancy regulator, are clustered within 23−25 Mbp on chromosome 7. The QTLs *qSD^S^-7-1* (Gu et al., 2004), *qSD7.1* (Wang et al., 2014), and *sdr4* (Sugimoto et al., 2010) are associated with seed dormancy, and *qMT-SGC 7.2* (Jiang et al., 2011) has been reported to be related to seed germination. The target trait of *qPHS-7* (Dong et al., 2003) was PHS. In this study, *qPH7* was not directly included in the loci mentioned above, except for *qMT-SGC7.2*, whose position was ambiguous because of the lack of information on a left flanking marker. In a practical sense, *Sdr4* was the most closely positioned target region of *qPH7* but was also approximately 11.6 Kbp away from *qPH7* to the right. These results indicate that the QTL *qPH7* could be considered a novel QTL conferring resistance to PHS derived from weedy rice sources.

Through fine mapping using CAPS marker sets, we were able to delimit the target region within a segment of approximately 210 Kbp flanked by the DNA markers *cPH7_7* and *7820873*, as well as 28 putative candidates ORFs involved in the region, based on the current gene annotation (**Supplementary Table S4**). To confirm the candidates, qPCR was performed using new primer sets designed based on exon sequences. We verified the expression of 15 of these ORFs and the expression levels of Os07g054366 in Wandoaengmi6, which were found to be significantly higher than those in Hwayeong (**Figure 5A**). However, the putative function of the gene Os07g054366 in rice is unknown. Moreover, the expression of this gene was found in panicles among other parts of rice, and was maintained in Wandoaengmi6 at a constant level during the treatment period for PHS screening (**Figure 5B**). The dormancy duration of Wandoaengmi6 (**Table 1**) suggests that the gene expression remained at a similar level for approximately a month post-harvest, decreasing thereafter.

The markers cPH7_7 for *qPH7* and cPH2_4 for *qPH2* were used to select promising lines with PHS resistance and other practical characteristics (**Supplementary Tables S3, S6**). Comparing the agronomic performance (blast resistance, germination rate, major agronomic traits, grain appearance, and yield) with the parents, three promising lines (HW20, HW23, and HW36) with both *qPH7* and *qPH2* were ultimately selected (**Table 3** and **Supplementary Table S5**). In particular, the line HW20 was used for marker-assisted selection or breeding (MAS or MAB) to improve the resistance of *japonica* cultivars to PHS.

## Conclusions

Coat-imposed dormancy, which resulted in resistance to PHS in the tested population, was found to cause resistance to PHS in Wandoaengmi6. Using QTL analysis, two QTLs, *qPH7* and *qPH2*, were associated with PHS resistance in rice using RILs derived from a cross between *japonica* weedy rice, Wandoaengmi6, and Hwayeong. In particular, from the fine mapping of the main QTL, *qPH7*, one ORF, Os07g054366, was found to be expressed significantly higher in Wandoaengmi6 than in Hwayeong, specific to the panicle. Thus, these results provide a reference for the improvement of PHS resistance in many other *japonica* cultivars without resistance using the PCR-based marker suggested in this study. In future studies, we plan to construct a sequence of the full-length cDNA of the gene detected herein, as well as perform functional analysis using transgenic plants.

## Data availability statement

The data presented in the study are deposited in the NCBI SRA repository, accession number PRJNA962984.

## Author contributions

C-ML wrote the original draft and evaluated all traits tested in this study, including QTL. M-KB developed the test population, evaluated the agronomic traits of the RILs, and advanced the population using SSD. O-YJ and JS provided critical feedback for data interpretation and contributed to writing the manuscript. H-SP was involved in all aspects of the study, reviewed the manuscript and the data, provided critical feedback for data interpretation, and contributed to writing the manuscript. S-MK designed the study and reviewed and edited the manuscript. All authors have read and approved the final manuscript.
