# Supplementary figures and images for: QTL mapping and improvement of pre-harvest sprouting resistance using japonica weedy rice
Source: Front Plant Sci. 2023 Jun 5;14:1194058. doi: 10.3389/fpls.2023.1194058 (PMC10277695; doi:10.3389/fpls.2023.1194058)

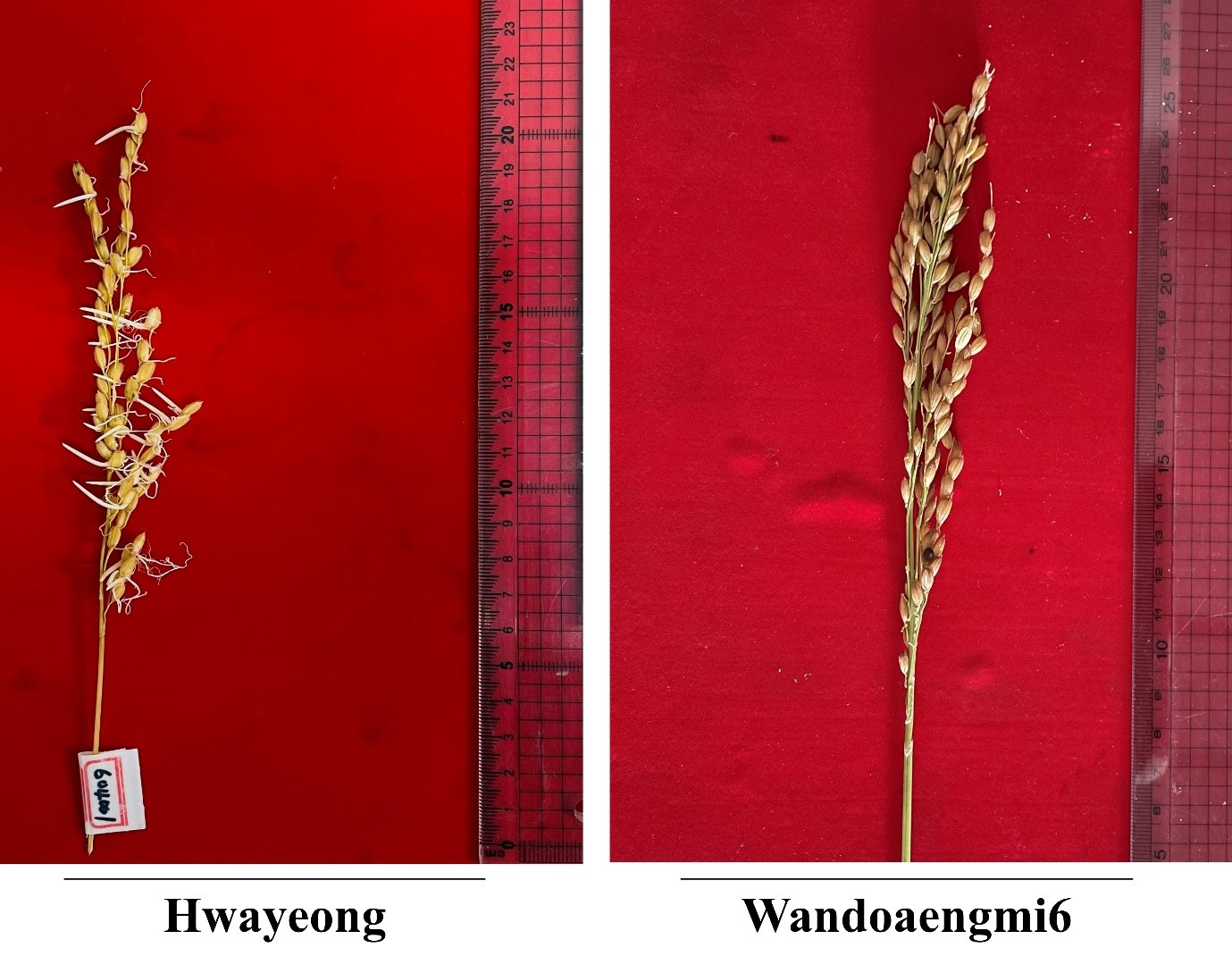

Supplement: Supplementary file 1 [file Image_1.jpeg]

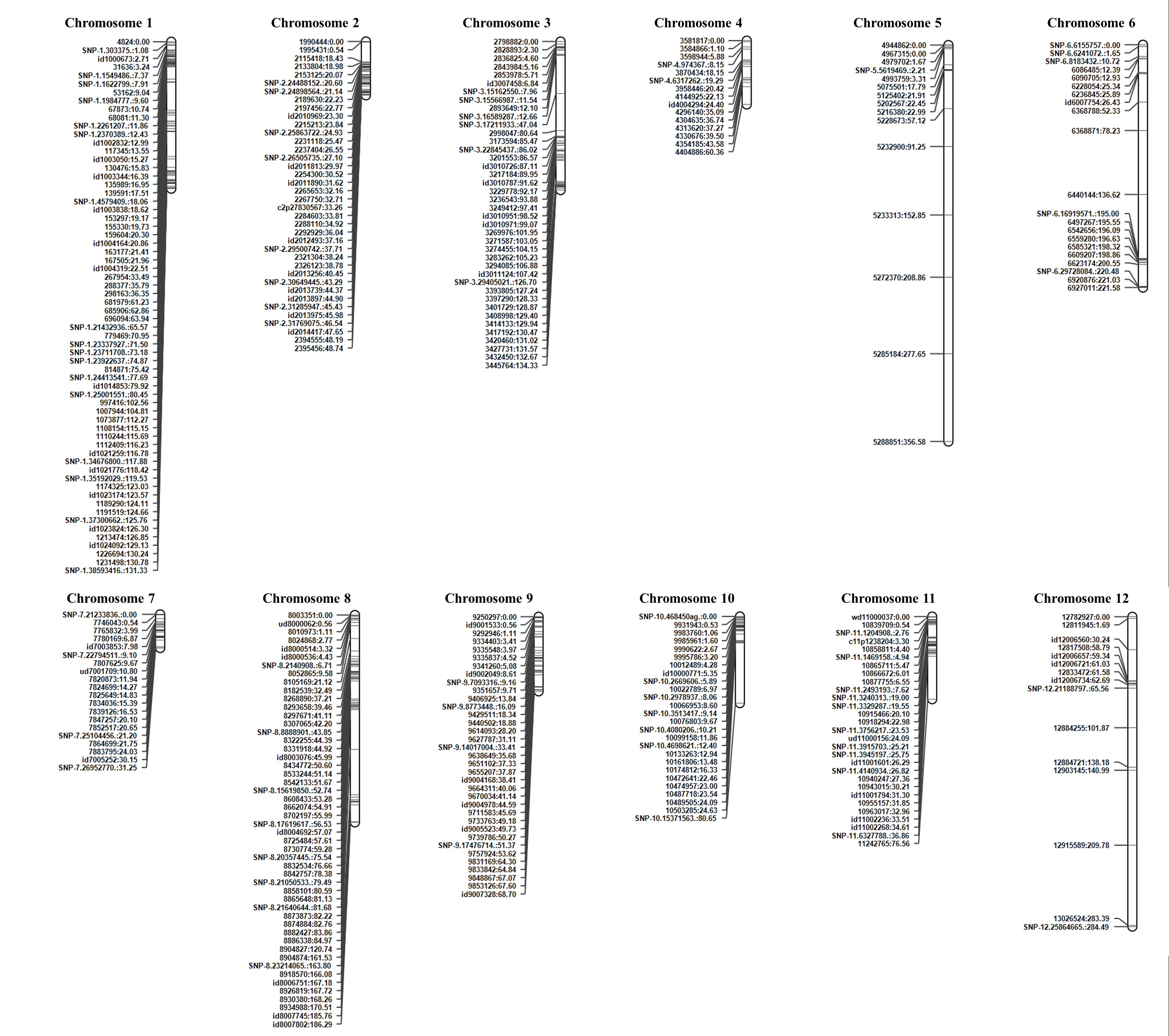

Supplement: Supplementary file 2 [file Image_2.jpeg]
